# Heavy Metal Levels in Adolescent and Maternal Blood: Association with Risk of Hypospadias

**DOI:** 10.1155/2014/714234

**Published:** 2014-03-04

**Authors:** Tusha Sharma, Basu Dev Banerjee, Chandra Shekhar Yadav, Piyush Gupta, Sunil Sharma

**Affiliations:** ^1^Environmental Biochemistry & Molecular Biology Laboratory, Department of Biochemistry, University College of Medical Sciences & GTB Hospital, University of Delhi, Dilshad Garden, Delhi 110095, India; ^2^Department of Pediatrics, University College of Medical Sciences & GTB Hospital, University of Delhi, Dilshad Garden, Delhi 110095, India; ^3^Department of Environmental Studies, Delhi University, Delhi 110007, India

## Abstract

*Background*. Hypospadias is a part of testicular digenesis syndrome (TDS) which includes infertility, cryptorchidism, and spermatogenesis. Heavy metals act as endocrine disrupting compounds. Heavy metals such as cadmium, chromium, arsenic, and lead have been associated with male infertility, cryptorchidism, spermatogenesis, cancer, reproductive disorder, and neurological disorder. However, it remains an important issue to corroborate or refute the hypothesis that the role of heavy metals in male reproductive tract disorders. Hence, the present study was designed to investigate the possible association of heavy metal and risk of hypospadias by estimating the blood heavy metal levels. *Methods*. In this case control study, 50 hypospadias boys diagnosed and confirmed by a pediatric urologist and 50 randomly selected age-matched (1–5 years) healthy control boys not suffering from any clinically detectible illness and their mothers have been included and heavy metal levels in the blood of these subjects have been estimated by Atomic Absorption Spectrophotometer (AAS). *Result*. Significantly high levels of cadmium and lead have been observed in hypospadias cases; however, all heavy metal levels were present in higher concentration. *Conclusion*. Higher blood levels of cadmium and lead may be associated with the increased risk of hypospadias.

## 1. Introduction

Hypospadias is urogenital birth defect and a part of testicular digenesis syndrome (TDS) which includes infertility, cryptorchidism, and spermatogenesis. The development of the male reproductive organs is controlled by sex hormones, and particular androgen plays a crucial role during the first trimester of pregnancy [1]. Fetal exposure to chemicals with antiandrogen or estrogen-like activity may interfere with normal hormonal signalling, which may increase the risk of hypospadias and other male reproductive disorders [2, 3]. Many chemicals including dioxins and furans, polychlorinated biphenyls, organochlorine pesticides, and some heavy metals have been identified as potential endocrine disrupters [4].

Exposure to heavy metals such as arsenic (As), chromium (Cr), cadmium (Cd), and lead (Pb) is a serious growing concern throughout the world. This exposure has risen dramatically in the last few decades due to an exponential increase in the use of these heavy metals in various industrial processes such as dyeing, metal refining, and plastic. Occupational exposure to heavy metals such as Cd, Cr, and Pb has been reported and has been associated with cancer, reproductive disorder, neurological disorders, and so forth. Worldwide, tons of toxic industrial wastes are mixed with liquid agricultural fertilizers and dispersed across farmlands. In general, heavy metals are systemic toxins with specific nephrotoxic, fetotoxic, and teratogenic effects.

Heavy metal toxicity has been associated with male infertility, reproductive disorder, reduced spermatogenesis, and so forth. Cadmium (Cd) is an environmental toxicant and acts as endocrine disruptor in human system. Several organs (e.g., kidney, liver, and testis) are affected by high levels of Cd. Cd in combination with other heavy metals such as Cr, As, and Pb may account for decreased male fertility rate in the developed countries observed by reduced sperm counts and testis function [5]. Chronic exposure to lead can also induce functional disorder (decrease of testosterone synthesis) or morphological disorder. Environmental and occupational exposure to lead have been associated with increasing number of diagnosed fertility impairments [6]. Chronic exposure to heavy metals such as arsenic may also affect sperm quality. Trace amounts of As have been observed in semen which suggests that it may inhibit the function of enzymes present in the acrosome and the membrane which covers the head of the sperm. However, it remains an important issue to corroborate or refute the hypothesis that the role of heavy metals in male reproductive tract disorders [7–15]. Hence, the present study was designed to investigate the possible association between extent of heavy metal exposure with the etiology of hypospadias with reference to heavy metal levels in blood of boys and their mothers and compare it with that of control cases.

## 2. Material and Method

### 2.1. Study Population

This case-control study was performed at the Department of Biochemistry, University College of Medical Sciences & GTB Hospital. Eligible cases were male children diagnosed with hypospadias without any concomitant malformation (cleft lip and/or plate, or cryptorchidism) or chromosomal abnormalities at the Department of Pediatrics, UCMS & GTB Hospital. In this study, 50 hypospadias boys diagnosed and confirmed by a pediatric urologist and 50 randomly selected age-matched (1–5 years) healthy control boys not suffering from any clinically detectible illness and their mothers have been included. A self-administered questionnaire was used to obtain relevant information including maternal age, maternal history of environmental/occupational exposure, and dietary habits (vegetarian/nonvegetarian). Information about birth weight was collected through infant medical records (Table 1). Informed consent was obtained from parents/guardians of all the subjects and the study was approved by the Institutional Ethics Committee for Human Research.

## 3. Digestion, Cleanup, and Quantification of Heavy Metals (As, Cr, Cd, and Pb) from Blood Samples

A total of 1.5 mL peripheral blood sample was collected in EDTA vial from subjects. About 0.5 mL of blood sample was taken in triplicate in 100 mL digestion flask fitted with 30 cm long air condenser and 5.0 mL distilled HNO_3 _was added to each sample. The contents were heated at 80°C for 30 min. After cooling, 1.5 mL of concentrated perchloric acid (70%) was added and sample was heated again at 250°C with occasional shaking till white fumes evolved. Clear solution was cooled and transferred into a 10 mL measuring flask. The volume was made up to 10 mL with deionized water (Rehman et al.). Thus, obtained sample was filtered by using syringe filter of 0.45 micron pore size (RFCL Ltd., New Delhi) and the metal levels were analyzed by Schimadzu Flame AA-6800 (Atomic Absorption Spectrophotometer) from Delhi University.

## 4. Statistical Analysis

All the values were expressed as mean ± SD along with percentile basis. Heavy metals levels in mothers and children were analyzed by *t*-test followed by Levene's test. Correlation between individual metals in maternal and child blood was tested using Pearson's coefficient of determination. The values *P* < 0.05 were considered statistically significant.

## 5. Result

Demographic profile of children and mothers in both cases and controls is given in Table 1. We found a significant positive association between low birth weight (<2500 gm) and hypospadias (*P* < 0.02). But we did not find any significant association of maternal age, occupational and environmental exposure, and dietary habit with hypospadias. Metal levels of hypospadias boys and controls and their mothers are listed in Tables 2 and 3, respectively. In hypospadias cases, maternal and child blood had higher and statistical significant levels of cadmium and lead than control cases. Although concentrations of chromium and arsenic were also found higher in hypospadias cases as compared to control cases in blood samples of mothers as well as their children, the differences were not statistically significant. In hypospadias cases, maternal and child blood had higher Pearson's coefficients in case of cadmium and lead, and this shows a statistically significant and positive correlation between maternal and child blood levels of cadmium and lead (Table 4).

## 6. Discussion

We found significantly higher concentration of cadmium, chromium, and lead in hypospadias cases than control group, which showed association between high levels of cadmium, chromium, and lead and risk of hypospadias. Nearly 70% of all birth defects have no known risk factors; therefore, attention to the risk of birth defects due to occupational exposure could be of great interest. Previous authors have addressed the birth defects related to limited exposures, such as pesticides, heavy metals, glycol ethers, and inhalation of anaesthetic drugs [16–19]. Furthermore, occurrence of hypospadias has been closely related with cryptorchidism, risk of testicular cancer, and possibly reduced semen quality [20].

A recent case-control study indicates that hypospadias may be associated with a reduced couple fertility which was corroborated in a recent case-control study, clearly corroborating a dose-related association between years of involuntary childlessness and the risk of hypospadias [21, 22]. Furthermore, authors have also suggested an association between maternal occupational exposure to endocrine disrupting chemical and risk of cryptorchidism in boys [23]. Benoff et al. (2000) have reported that heavy metals are associated with male infertility and reproductive disorder. They have also reported that infertility, low serum count, sperm motility, and increased number of spermatozoa with morphological abnormalities may be associated with cadmium and lead toxicity. They have also suggested that occupational exposure to cadmium is associated with increased risk of prostate cancer and prostate neoplasm [24]. The common source of cadmium exposure is through rice grown in cadmium contaminated water. Occupational exposure to cadmium is through industries involved in metal refining, batteries, and tobacco.

In this study, we have observed high levels of heavy metals in mothers as well as in children and found a positive association between maternal and child blood levels of cadmium and lead in hypospadias. However, the mechanism behind this association is not clear, but it has been suggested that, due to endocrine disrupting potential of cadmium and lead, they may cause cryptorchidism, male infertility, and risk of testicular cancer in males which are the components of the TDS. Studies have reported that both cadmium and lead may transfer from mother to child through placenta and reach the embryo during early gestation; Cd disturbs the placental function and also causes secondary foetal damage. Occupational exposure to cadmium has been associated with various tumours in multiple organs including lungs, prostate, and testis. Lead affects both male and female reproductive systems. In men, when blood lead level exceeds 40 *μ*g/dL, a reduction in sperm count, volume of sperm, their motility, and altered morphology has been reported. Pregnant woman's elevated blood lead levels have been associated with miscarriage, prematurity, low birth weight, and fetus development [25]. *In utero* exposure to lead from the mother's bones may act as toxin for fetus. Breast milk and blood lead levels in mothers may also effect child development [26]. Johnson et al. [27] and Moon [28] have also reported increased blood lead levels in the children age group 2–10 years. Similarly, we have also observed high levels of cadmium and lead in both mother and child.

In conclusion, our results suggest that higher blood levels of cadmium and lead may be associated with the increased risk of hypospadias. However, small sample size limits the sensitivity and statistical power of our study. Hence, these results must be interpreted with caution. Larger studies on heavy metals exposure and risk of hypospadias keeping in view molecular target or mechanism of disease etiology are further required to address these uncertainties.

## Figures and Tables

**Table 1:** Demographic profile of mothers and their children.

Characteristics	Cases (*n* = 50)	Controls (*n* = 50)	*P* value
Maternal age at delivery			
≥30 years	25 (50%)	25 (50%)	1.00
30 years	25 (50%)	25 (50%)	
Maternal h/o environmental/occupational exposure			
Yes	31 (63.3%)	6 (13.3%)	0.07
No	19 (36.6%)	44 (86.6%)	
Dietary habits			
Nonvegetarian	36 (73.3%)	23 (46.6%)	0.18
Vegetarian	14 (26.6%)	27 (53.3%)	
Child birth weight (in gm)			
<2500	28 (56.6%)	3 (6.6%)	0.02*
Normal (≥2500)	22 (43.3%)	47 (93.3%)	

*Significant at the *P* < 0.05 level.

**Table 2:** Comparison of heavy metal levels (ppm/mL) in blood of cases and control mothers.

Heavy metals	Cases (*n* = 50)				Controls (*n* = 50)				*P* value
	Mean ± SD	25%	50%	75%	Mean ± SD	25%	50%	75%	
Cadmium	1.19 ± 0.93	0.64	1.06	1.32	0.08 ± 0.26	0.00	0.01	0.06	0.01*
Chromium	0.80 ± 0.57	0.42	0.61	1.11	0.29 ± 0.49	0.08	0.17	0.27	0.01*
Arsenic	3.43 ± 2.31	1.12	3.36	4.67	2.22 ± 4.03	0.03	0.20	2.73	0.11
Lead	3.51 ± 4.93	0.14	0.50	6.86	1.04 ± .889	0.66	0.84	1.28	0.01*

*Significant at the *P* < 0.05 level.

**Table 3:** Comparison of heavy metal levels (ppm/mL) in blood of hypospadias and control boys.

Heavy metals	Cases (*n* = 50)				Controls (*n* = 50)				*P* value
	Mean ± SD	25%	50%	75%	Mean ± SD	25%	50%	75%	
Cadmium	1.21 ± 1.12	0.39	0.84	1.60	0.48 ± 0.67	0.05	0.09	0.64	0.01*
Chromium	0.72 ± 0.99	0.09	0.33	1.06	0.66 ± 0.42	0.31	0.65	0.99	0.75
Arsenic	1.80 ± 1.26	0.86	1.459	2.78	1.42 ± 1.22	0.65	1.01	2.24	0.49
Lead	3.09 ± 3.55	0.69	1.17	5.30	0.12 ± 0.21	0.04	0.06	0.10	0.01*

*Significant at the *P* < 0.05 level.

**Table 4:** Pearson's correlation coefficient (*r*) for metal in maternal and child blood in control and hypospadias cases.

Heavy metals	Control	Cases
Cadmium	0.86	0.94*
Chromium	0.97	0.94
Arsenic	0.90	0.07
Lead	0.77	0.86*

*Correlation is significant at the *P* < 0.05 level.
